# Traditional Chinese herbs and natural products in hyperuricemia-induced chronic kidney disease

**DOI:** 10.3389/fphar.2022.971032

**Published:** 2022-08-09

**Authors:** Letian Yang, Bo Wang, Liang Ma, Ping Fu

**Affiliations:** Kidney Research Institute, Division of Nephrology, West China Hospital of Sichuan University, Chengdu, China

**Keywords:** chronic kidney disease, hyperuricemia, hyperuricemic nephropathy, herbal medicine, natural product

## Abstract

Hyperuricemia is a common biochemical disorder, which resulted from both excessive uric acid (UA) production and/or absolute or relative impairment of urinary UA excretion. Growing evidence has indicated that hyperuricemia is an independent risk factor for the development and progression of chronic kidney disease (CKD), causing hyperuricemia-induced CKD (hyperuricemic nephropathy, HN). The therapeutic strategy of HN is managing hyperuricemia and protecting kidney function. Adverse effects of commercial drugs make persistent treatment of HN challenging. Traditional Chinese medicine (TCM) has exact efficacy in lowering serum UA without serious adverse effects. In addition, TCM is widely applied for the treatment of CKD. This review aimed to provide an overview of efficacy and mechanisms of traditional Chinese herbs and natural products in hyperuricemia-induced CKD.

## 1 Introduction

Hyperuricemia is a common biochemical disorder, which resulted from both excessive uric acid (UA) production and/or absolute or relative impairment of urinary UA excretion. According to previous experiences, hyperuricemia is defined as persistent serum UA concentrations of > 7 mg/dl (>420 μmol/L) in men and > 6 mg/dl (>360 μmol/L) in women (Johnson et al., 2018). With the improvement of the economic level and the change in people’s lifestyle and dietary structure, the global incidence and prevalence of hyperuricemia tend to increase steadily. Based on the findings from two nationally representative cross-sectional surveys in 2015–16 and 2018–19, the estimated prevalence of hyperuricemia among Chinese adults is 11.1% (Zhang et al., 2021).

It is already observed that urate in the crystal form could deposit in joints and tissues. The deposition maybe asymptotic at first; then clinical manifestations such as arthralgia, hypertension, abnormal glucose tolerance, and renal dysfunction occur gradually (Grassi et al., 2013). Previous studies have proven that hyperuricemia is an independent risk factor for the development and progression of chronic kidney disease (CKD) (Johnson et al., 2018; Pan et al., 2021). Growing numbers of evidence has indicated that elevated serum UA caused hyperuricemic nephropathy (HN), presented with uric acid–related kidney stones, renal obstruction, and acute or chronic renal dysfunction (Pan et al., 2019; Pan et al., 2021).

The strategy of HN treatment is managing hyperuricemia and protecting renal function. Several commercial UA-lowering drugs have been widely used in clinics, such as allopurinol, febuxostat, and benzbromarone. However, the adverse effects limited their application. Long-term clinical practice has demonstrated that traditional Chinese medicine (TCM) has exact efficacy in lowering serum uric acid without serious adverse effects (Sun et al., 2015). When it comes to renal-protective effects, TCM is widely applied for the treatment of CKD, such as *Abelmoschus manihot* (Huangkui), *Cordyceps*, and Danshen (Shao et al., 2021).

This review aimed to provide an overview of efficacy and mechanisms of traditional Chinese herbs and extracted natural products in hyperuricemic nephropathy.

## 2 Serum uric acid regulation

The serum uric acid consists of the production and excretion. If the homeostasis of serum UA is demolished, patients will suffer from hyperuricemia (Gliozzi et al., 2016).

### 2.1 Production of uric acid

UA, produced in the liver, is the end-product of the metabolic pathway of purine nucleic acids; degradation of proteins and fructose metabolism also play important roles in generating uric acid (Sato et al., 2019). Xanthine oxidoreductase (XOR) is an enzyme with dehydrogenase activity. It catalyzes the last two steps of purine catabolism, the conversion of hypoxanthine to xanthine and xanthine to UA (Chen et al., 2016a). XOR is mainly present in the liver and also found in the intestines, gastrointestinal tract, muscle, and blood vessels (Chen et al., 2016a; Battelli et al., 2016).

### 2.2 Excretion of uric acid

Urate is freely filtered at the renal glomerulus, and most of the filtered urate is reabsorbed in the renal tubules. The reabsorption and excretion of UA in the kidney, mediated by rate reabsorption transporters and urate excretion transporters located in the renal tubular epithelium, are responsible for the metabolic balance of UA (VanWert et al., 2010; Xu et al., 2017; Li et al., 2019). Urate reabsorption transporters consist of urate anion transporter 1 (URAT1), organic anion transporter 4 (OAT4), and glucose transporter 9 (GLUT9) (Nigam et al., 2015; Benn et al., 2018). Urate excretion transporters mainly have four members: organic anion transporter 1 (OAT1), organic anion transporter 4 (OAT3), multidrug resistance protein 4 (MRP4/ABCC4), and ATP-binding cassette superfamily G member 2 (ABCG2) (VanWert et al., 2010; Pena-Solorzano et al., 2017; Benn et al., 2018). Because of lack of uricase in the human body, UA cannot convert to allantoin with high solubility in water.

## 3 Mechanism of hyperuricemic nephropathy

The mechanism of HN is complex. The conventional view holds that hyperuricemia causes CKD due to the deposition of urate crystals in the renal tubules. However, growing evidence has indicated that uric acid could induce kidney damage through crystal-independent mechanisms.

### 3.1 Inflammation

Previous studies have reported that hyperuricemia could induce renal inflammation. Uric acid could increase the expressions of monocyte chemotactic protein-1 (MCP-1), which is known as a pro-inflammatory factor (Baldwin et al., 2011). Roncal et al. (2007) conducted a cisplatin-induced acute kidney injury mouse model and indicated that uric acid exacerbated renal injury *via* a pro-inflammatory pathway. In addition, uric acid activated the renal tubular NF-κB signaling pathway, thus inducing renal inflammation (Zhou et al., 2012).

### 3.2 Oxidative stress

A large number of studies have confirmed that oxidative stress and secondary injury of endothelial cells are contributors to the pathophysiology of CKD. Hyperuricemia induced intrarenal oxidative stress *via* increasing the expression of NADPH oxidase 4 (NOX-4) and angiotensin II (Sánchez-Lozada et al., 2008). Furthermore, serum uric acid could decrease nitric oxide (NO) bioavailability, leading to the injury of endothelia cells (Sánchez-Lozada et al., 2008). Notably, during production of uric acid, numerous numbers of reactive oxygen species (ROS) are generated, which significantly affect the endothelial function.

### 3.3 Fibrosis

Renal fibrosis is one of the main pathological changes of CKD. Uric acid could increase the expressions of intercellular cell adhesion molecule-1 (ICAM-1) and vascular cell adhesion molecule-1 (VCAM-1), resulting in renal interstitial fibrosis (Zhou et al., 2012). In addition, uric acid could activate several intracellular profibrotic signaling pathways, including the TGF −β1 pathway, ERK1/2 pathway, PI3K/Akt pathway, and JAK/STAT pathway (Lyngdoh et al., 2011).

## 4 Commercial drugs for hyperuricemic nephropathy

The key to long-term management of hyperuricemia is maintaining the serum uric acid under the saturation level (Gliozzi et al., 2016). So far lowering uric acid mainly focuses on two targets, XOR and renal urate transporters. Since XOR is a critical enzyme in purine catabolism, it is a significant target of uric acid–lowering drugs. Commercial XOR-inhibitor drugs include allopurinol, febuxostat, and topiroxostat (Chen et al., 2016a). XOR-inhibitor drugs are viewed as the primary urate-lowering therapy (Gliozzi et al., 2016). However, their adverse effects limit the clinical use. It has been reported that allopurinol is associated with fatal bone marrow depression and hepatotoxicity (Chen et al., 2016a; Stamp et al., 2016). Impaired liver function is the most common adverse event of febuxostat (Chen et al., 2016a; Jordan and Gresser, 2018). In addition, clinicians also find febuxostat could result in serious hypersensitivity reactions such as Stevens–Johnson syndrome (SJS) and a higher incidence of Antiplatelet Trialists’ Collaboration (ATPC) events compared to allopurinol (Becker et al., 2010; Chen et al., 2016a). Currently, the safety of topiroxostat has been proven in animals. However, because topiroxostat is only shortly used in Japan, international clinical trials are needed to investigate its effects and safety (Chen et al., 2016a; Sezai et al., 2017). Uricosuric drugs could be used if XOR inhibitor does not work (Gliozzi et al., 2016). Benzbromarone is a urate transport inhibitor mainly inhibiting URAT1 in humans. Benzbromarone hepatotoxicity, such as liver dysfunction and serious hepatitis, limits its clinical use (Gliozzi et al., 2016; Strilchuk et al., 2019). Notably, uricosuric drugs are not appropriate for patients with impaired kidney function (eGFR < 20 ml/min) (Bach and Simkin, 2014; Vargas-Santos and Neogi, 2017).

## 5 UA-lowering effects of traditional Chinese herbs and extraction of natural products for hyperuricemic nephropathy

**FIGURE 1:**
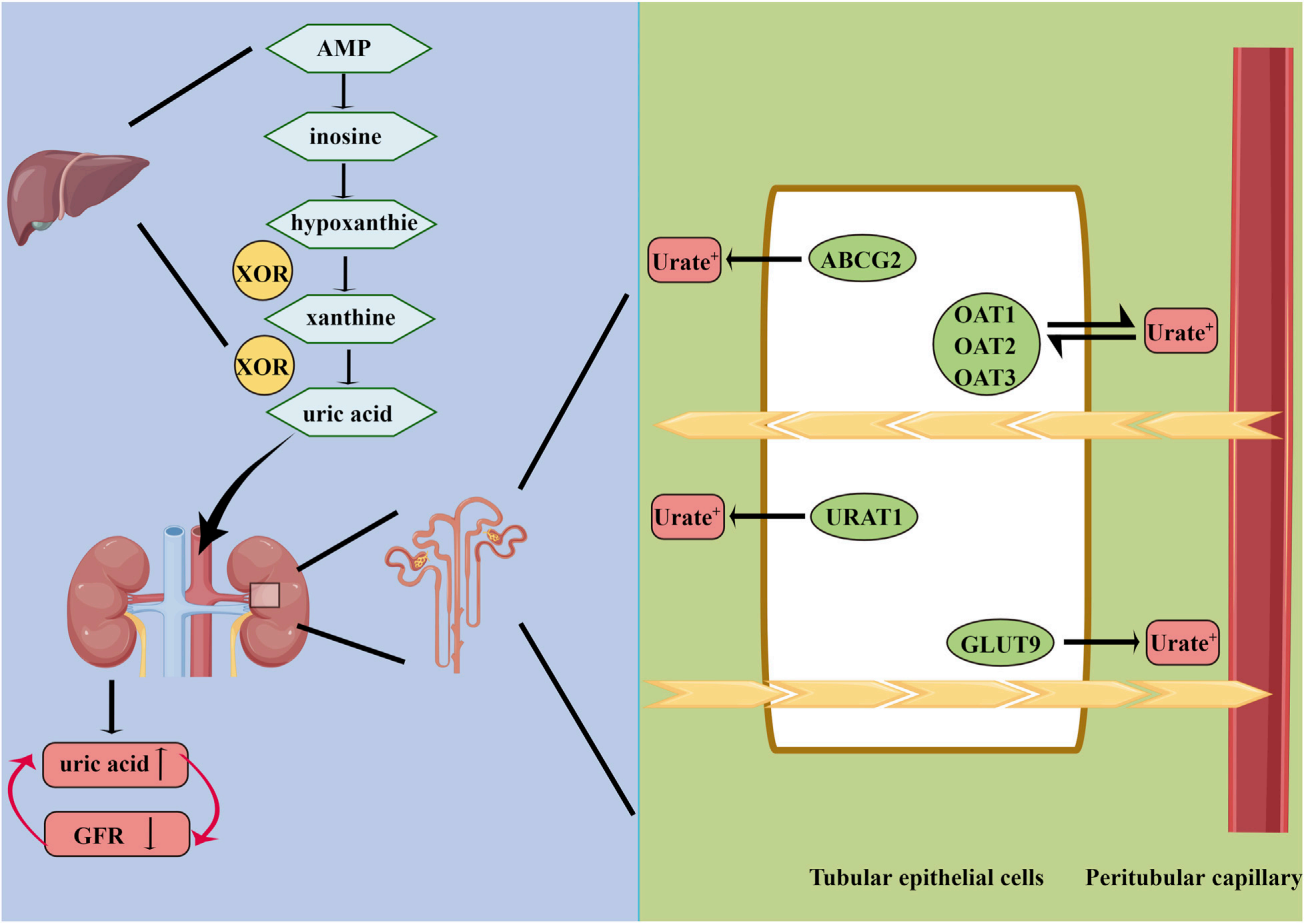
Summary of uric acid metabolism. AMP, adenosine monophosphate; XOR, xanthine oxidoreductase; GLUT9, glucose transporter 9; OAT1, organic anion transporter 1; ABCG2, ATP-binding cassette superfamily G member 2; URAT1, urate anion transporter 1; OAT3, organic anion transporter 3; GFR, glomerular filtration rate.

In the TCM theory, hyperuricemia results from dysfunction of the spleen and kidney (Chen et al., 2016b; Huijuan et al., 2017). Most patients are overweight and have a predilection for oily food. Unhealthy living habits lead to disorders of viscera, causing blood stasis with water retention, and dampness–heat pouring downward (Kong et al., 2004; Yu et al., 2018). Multiple Chinese herbs and formulas, aiming to clear heat and drain dampness, have been proven effective and safe in the treatment of hyperuricemia (Yu et al., 2018). In recent years, modern pharmacological studies have conducted the HN animal model to verify the protective effects of several traditional Chinese herbs, including UA-lowering effects and renal-protective effects. For lowering UA, there are two targets (liver XOR and renal urate transporters), similar to commercial drugs (Figure 1). The UA-lowering effects and related targets are summarized in Table1.

**TABLE 1:** Uric acid–lowering effects of traditional Chinese herbs.

Traditional Chinese herb	Animal model	UA-lowering targets	Reference
*Smilax glabra* (*Tu-Fu-Ling*)	HN mice	XOR, GLUT9, and OAT1	(Chen et al., 2011; Li et al., 2017; Shi et al., 2018; Yan et al., 2018)
	HN rats		
*Smilax china* L. (*Ba-Qia*, *Jin-Gang-Teng*)	HN mice	XOR	Meng et al. (2017)
Mesona procumbens Hemsl. (*Xian-Cao*)	diabetic rats	XOR, GLUT9, and OAT1	Chen et al. (2020)
	HN mice		
Scutellariae radix (*Huang-Cen*)	HN mice	XOR	(Dong et al., 2017; Bao et al., 2018; Hua et al., 2018)
*Morus alba* L. (*Sang-Shu*)	HN mice	XOR, URAT1, GLUT9, and OAT1	(Yang et al., 2008a; Yang et al., 2008b; Chan et al., 2016; Jhang et al., 2016; Phimarn et al., 2017; Yeh et al., 2019)
*Rhododendron oldhamii* Maxim. (*Zhuan-Hong-Du-Juan*)	HN mice	Unclear	Hunyadi et al. (2013)
*Chrysanthemum morifolium* Ramat. (*Ju-Hua*)	HN rats	XOR, ABCG2, URAT1, and GLUT9	Caselli et al. (2016)
*Liriodendron chinense* (Hemsl.) Sarg (*E-Zhang-Qiu*)	HN mice	OAT1, OAT3, and ABCG2	Sun et al. (2015)
Fructus Gardenia (*Zhi-Zi*)	HN mice	XOR, URAT1, GLUT9, OAT1, and OAT3	(Peng et al., 2019; Liu et al., 2020)
Poria cocos (*Fu-Ling*)	HN mice	ABCG2	Liu et al. (2017)
Dendrobium officinalis six nostrum (*Tie-Pi-Shi-Hu*)	HN rats	ABCG2 and GLUT9	Tung et al. (2015)

HN, hyperuricemic nephropathy; XOR, xanthine oxidoreductase; GLUT9, glucose transporter 9; OAT1, organic anion transporter 1; ABCG2, ATP-binding cassette superfamily G member 2; URAT1, urate anion transporter 1; OAT3, organic anion transporter 3.

### 5.1 Inhibition of the liver xanthine oxidoreductase activity

#### 5.1.1 *Smilax china* L

*Smilax china* L., also known as “*Ba-Qia*” (or “*Jin-Gang-Teng*”) in China, is a well-known traditional Chinese herb. It has been widely used in treatment of gout and rheumatoid arthritis (Chen et al., 2011). At present, several bioactive compounds have been isolated and identified from *Smilax china* L., such as flavonoids, polyphenols, steroidal saponins, and polysaccharides (Li et al., 2022). Chen et al. (2011) found that five fractions (petroleum ether, chloroform, ethyl acetate, n-butanol, and residual ethanol fraction) of *Smilax china* L. could significantly lower serum UA in HN mice. Moreover, *in vitro* studies have indicated that the aforementioned five fractions could markedly inhibit the liver activity of XOR.

#### 5.1.2 Scutellariae radix

Scutellariae radix (named *Huang-Cen* in China) is widely used in Chinese folk formulas to reduce uric acid and treat gout. Baicalein is a natural flavonoid extracted from Scutellariae radix, which has great effects to treat inflammation, cancer, hepatic disorder, neuronal damage, and cardiovascular diseases (Li et al., 2017; Shi et al., 2018; Yan et al., 2018; Zhou et al., 2018). Meng et al. (2017)havefound that baicalein treatment could significantly suppress the viability of XOR in the HN mouse model (Meng et al., 2017). Hayashi et al. (1988) and Li et al. (2014a)also proved that baicalein possessed a strong effect to inhibit liver XOR activity.

### 5.2 Regulation of renal urate transporters

#### 5.2.1 *Liriodendron chinense* (Hemsl.) Sarg

*Liriodendron chinense* (Hemsl.) Sarg (named *E-Zhang-Qiu* in Chinese) belongs to the Magnoliaceae family mainly distributed in East Asia. *Liriodendron chinense* (Hemsl.) Sarg is widely used in China to treat rheumatic fever, rheumatoid arthritis, and osteoarthropathy (Li et al., 2014b; Pan et al., 2021). In TCM theory, the barks of *Liriodendron chinense* (Hemsl.) Sarg have been proven to have good effects on gout. Pan et al. (2021)observed that the ethanol extract of the barks of *Liriodendron chinense* (Hemsl.) Sarg could significantly lower serum UA levels *via* upregulating renal OAT1, OAT3, and ABCG2 proteins.

#### 5.2.2 Poria cocos

Poria cocos (named *Fu-Ling* in Chinese) is a classical TCM. As recorded in the Chinese Pharmacopoeia, Poria cocos have strong diuretic effects, widely used in treatment of edema, insomnia, and dyspepsia. Liang et al. (2021) reported that Poria cocos had excellent hypouricemic effects in HN mice and could remarkably elevate the expressions of renal ABCG2.

#### 5.2.3 Dendrobium officinalis six nostrum

Dendrobium officinalis six nostrum (named *Tie-Pi-Shi-Hu* in Chinese) is widely used in TCM to regulate blood sugar and enhance immunity. Chen, X. et al. verified that oral administration of Dendrobium officinalis six nostrum could obviously lower serum UA levels in HR rats *via* regulating expressions of renal ABCG2 and GLUT9 (45).

### 5.3 Both inhibit liver xanthine oxidoreductase activity and regulate renal urate transporters

#### 5.3.1 Fructus Gardenia

Fructus Gardenia (named *Zhi-Zi* in Chinese) is widely distributed throughout China. As a widely used traditional Chinese herb, Fructus Gardenia showed good treatment effects on hepatitis, hypertension, and diabetes (Ni et al., 2006; Liu et al., 2013). TCM considers that Fructus Gardenia has the functions of clearing heat and diuresis (Ni et al., 2006). Hu et al. (2013)found that extracts of Fructus Gardenia could significantly reduce serum UA levels in HN mice by regulating renal URAT1, GLUT9, OAT1, and OAT3 expressions. Geniposide is a key active ingredient in the fruits of Fructus Gardenia. A recent study showed that geniposide had a strong antihyperuricemia effect in HN mice by inhibiting liver XOR activity (Chen et al., 2022).

#### 5.3.2 Smilax glabra

*Smilax glabra* usually grows on the hillside, near river, or under forests, mainly distributed in Southwest China, including the Yunnan and Sichuan provinces. The rhizome of *Smilax glabra* is named *Tu-Fu-Ling* in China, has a long history of cultivation in east and Southeast Asia, and is widely used for detoxification, anti-inflammation, analgesia, diuresis, and antitumor activity (Dong et al., 2017; Bao et al., 2018; Hua et al., 2018). Chemical components of *Smilax glabra* were initially investigated in 1993. Today nearly 200 components have been named, most of which are extracted from *Tu-Fu-Ling*. Flavonoids are the most famous components among them. According to the Chinese Pharmacopoeia 2015 edition, astilbin (a flavonoid glycoside), 3,3,4’,5,7-pentahydroxyflavanone 3–6 [-deoxy ([alpha]-L-mannopyranoside)], is used to determine the content of *Smilax glabra* (Hua et al., 2018). It is well acknowledged that astilbin has great immunosuppressive and anti-inflammatory effects. *Smilax glabra* is an essential component of several famous Chinese formulas to treat gout, such as *Qi-Zhu-Xie-Zhuo-Fang* (Huijuan et al., 2017), *Xie-Zhuo-Chu-Bi-Fang* (Sun et al., 2015), and *Qu-Zhuo-Tong-Bi* decoction (Chen et al., 2016b). Ji, W. et al. conducted a clinical study in patients with repeatedly attacking acute gouty arthritis to investigate the effects and adverse events of *Re-Bi-Xiao* granules (a TCM consisting of Hypoglauca yam, giant knotweed rhizome, Phellodendron bark, *Smilax glabra* rhizome, etc.). The results showed good effects of this compound to treat gout. No serious adverse events were observed. The following animal study revealed that both compounds and *Tu-Fu-Ling* could significantly reduce uric acid levels (Ji et al., 2005). Liu et al. (2015) proved compound *Tu-Fu-Ling* granules could lower serum uric acid levels by downregulating renal GLUT9 in a hyperuricemic mouse model (Liu et al., 2015). In recent years, the hypouricemic effects of astilbin have gradually caused concerns (Dong et al., 2017). Wang et al. (2019) treated uric acid nephropathy rats with the flavonoid-rich fraction extracted from *Tu-Fu-Ling*. These extracts could remarkably decrease urine uric acid levels. Huang et al. (2019) isolated four astilbin stereoisomers from *Smilax glabra* using HPLC analysis. Astilbin had notable effects of reducing serum uric acid levels. Further investigation also showed that astilbin could suppress the activity of XOR and increase the protein content of renal OAT.

#### 5.3.3 *Mesona procumbens* Hemsl

*Mesona procumbens* Hemsl., called *Xian-Cao* in China, is an annual herb mainly used as ingredients of drinks and desserts in coastal areas of China. It is widely used in Chinese folk medicine to treat joint pain, lower blood pressure, and anti-inflammation. Recent pharmacological research studies also reported that *Mesona procumbens* Hemsl. took effects on protection of hepatic cell, myocardium, and renal tissue (Yang et al., 2008a; Yang et al., 2008b; Yeh et al., 2019). Jhang, J.J. et al. investigated the effects of *Mesona procumbens* Hemsl. on uric acid metabolism in potassium oxonate (PO)–challenged ICR mice and streptozotocin (STZ)-induced diabetic rats. Fifty percent of ethanol extracts could remarkedly decrease serum UA levels in these two animal models. In addition, 50% ethanol extract obviously inhibits the liver XOR activity in STZ-induced diabetic rats, which is less effective than allopurinol. An *in vitro* study also proved its XOR inhibitory effects. Moreover, these extracts could regulate the expressions of renal GLUT9 and OAT1 in STZ-induced diabetic rats (Jhang et al., 2016).

#### 5.3.4 *Morus alba* L

*Morus alba* L., commonly named mulberry or *Sang-Shu*, is native to central and northern China. The leaves, fruits, and roots of *Morus alba* L. have been used in TCM dating back to 2000 years ago (Chan et al., 2016). *Morus alba* L. has several bioactivities, such as antioxidant properties, antimicrobial activity, antidiabetic properties, anti-obesity activity, anti-inflammatory activity, and antihyperuricemia activity (Chan et al., 2016; Phimarn et al., 2017; Yuan and Zhao, 2017; Yuvaraj and Geetha, 2018). Recently, several flavonoids, alkaloids, phenolic acids, and coumarins in *Morus alba* L. have been identified. Ramulus Mori is the dried twigs of *Morus alba* L, which is found to have excellent hypouricemic effects. The ethanol extract of Ramulus Mori could enhance the excretion of uric acid by regulating the expressions of renal URAT1, GLUT9, and OAT1. An animal experiment conducted in Hungary investigated metabolic effects of mulberry leaves. Extracts of mulberry leaves exerted antihyperuricemic actions as potential uricosuric agents. *In vivo* and *in vitro* studies both proved its XOR inhibitory activities (Hunyadi et al., 2013). Yao et al. (2019) also proved the hypouricemic effects of Ramulus Mori refined extract (Yao et al., 2019). Several chemical compounds isolated from Ramulus Mori could take effects on treating gout and hyperuricemia. Morin is a well-known flavonoid isolated from twigs of *Morus alba* L., which is widely used as a yellow dye. Both *in vivo* and *in vitro* studies have indicated that it possessed numerous pharmacological activities, such as antioxidant activities and inhibition activity of XOR (Caselli et al., 2016; Sinha et al., 2016). Yu et al. (2006) indicated that morin could significantly increase the excretion of uric acid *via* inhibiting the urate uptake in renal brush border membrane vesicles, and the inhibition effect was much stronger than that of probenecid. Furthermore, morin also showed the XOR inhibitory effect. In addition, mulberroside A is a major stilbene glycoside isolated from *Morus alba* L. Wang et al. (2011) found that mulberroside A could lower serum uric acid in mice with hyperuricemia, which is attributed to decrease the expressions of renal GLUT9 and URAT1.

#### 5.3.5 *Chrysanthemum morifolium* Ramat

*Chrysanthemum morifolium* Ramat. (*Ju-Hua* in China) is one of the classical TCM and widely applied in the treatment of gout , chronic pain, dementia, and flu. Recent pharmacological studies have extracted a number of bioactive components, such as triterpenoids, flavonoids, volatile oils, and organic phenolic acids (Liu et al., 2020). Peng et al. (2019) have found that extracts of *Chrysanthemum morifolium* Ramat. significantly reduced serum UA levels in potassium oxonate–induced HN rats *via* inhibiting the liver XOR activity and regulating renal uric acid transport–related protein (ABCG2, URAT1, and GLUT9) expressions.

### 5.4 Others

*Rhododendron oldhamii* Maxim. (named *Zhuan-Hong-Du-Juan* in Chinese) is mainly distributed in Taiwan, China. The genus *Rhododendron* is widely used in TCM to treat arthritis, gout, asthma, and metabolic disorders. Nowadays, a large number of phenolic compounds have been isolated and identified from the genus *Rhododendron* (Liu et al., 2017). Tung et al. (2015)extracted four phenolic compounds from *Rhododendron oldhamii* Maxim. leaves, namely, (2R, 3R)-astilbin, hyposide, guaijaverin, and quercitrin. These four bioactive components could significantly lower serum UA levels in HN mice. But the mechanisms of its UA-lowering effect remain unknown.

## 6 Renal-protective effects of traditional Chinese herbs and extracted natural products for hyperuricemic nephropathy

In addition to UA-lowering effects, the aforementioned 11 traditional Chinese herbs and extracted natural products have renal-protective effects. For renal protection, researchers always used several markers to assess kidney damage, including serum creatinine, blood urea nitrogen (BUN), and renal pathology staining. In addition, several studies also explored the potential mechanisms of renal-protective effects, such as the inhibition of renal inflammation and fibrosis. The renal-protective effects and underlying mechanisms are summarized in Table2.

**TABLE 2:** Renal-protective effects of traditional Chinese herbs.

Traditional Chinese herb	Animal model	Renal-protective effect	Underlying mechanism	Reference
*Smilax glabra* (*Tu-Fu-Ling*)	HN rats	sCr, BUN renal pathology	Inhibit renal oxidative stress and inflammation	(Shi et al., 2018; Zhou et al., 2018)
*Smilax china* L. (*Ba-Qia*, *Jin-Gang-Teng*)	HN mice	BUN renal pathology	Unclear	Meng et al. (2017)
*Mesona procumbens* Hemsl. (*Xian-Cao*)	Diabetic rats	No change	Inhibit renal inflammation	Chen et al. (2020)
	HN mice			
Scutellariae radix (*Huang-Cen*)	HN mice	BUN renal pathology	Inhibit renal oxidative stress and fibrosis	Hua et al. (2018)
*Morus alba* L. (*Sang-Shu*)	HN mice	sCr, BUN	Unclear	(Phimarn et al., 2017; Yuvaraj and Geetha, 2018)
*Rhododendron oldhamii* Maxim. (*Zhuan-Hong-Du-Juan*)	HN mice	sCr, BUN renal pathology	Unclear	Hunyadi et al. (2013)
*Chrysanthemum morifolium* Ramat. (*Ju-Hua*)	HN rats	sCr, BUN	Inhibit renal inflammation	Caselli et al. (2016)
*Liriodendron chinense* (Hemsl.) Sarg (*E-Zhang-Qiu*)	HN mice	sCr, BUN renal pathology	Inhibit renal Inflammation and fibrosis	Sun et al. (2015)
Fructus Gardenia (*Zhi-Zi*)	HN mice	sCr, BUN renal pathology	Inhibit renal inflammation and fibrosis	(Peng et al., 2019; Liu et al., 2020)
Poria cocos (*Fu-Ling*)	HN mice	sCr, BUN renal pathology	Unclear	Liu et al. (2017)
Dendrobium officinalis six nostrum (*Tie-Pi-Shi-Hu*)	HN rats	sCr	Inhibit renal inflammation	Tung et al. (2015)

HN, hyperuricemic nephropathy; sCr, serum creatinine; BUN, blood urea nitrogen.

### 6.1 Inhibition of renal inflammation

Among the aforementioned 11 traditional Chinese herbs, six herbs or their extracted natural products could inhibit renal inflammation. The barks of *Liriodendron chinense* (Hemsl.) Sarg have good renal-protective effects *via* inhibiting renal inflammation through NF-κB and ASK1/JNK/c-Jun signaling pathways in HN mice (Pan et al., 2021). Dendrobium officinalis six nostrum significantly decreased renal inflammatory factors in HN rats (Chen et al., 2020). Geniposide extracted from Fructus Gardenia could protect renal function in HN mice *via* inhibiting inflammation (Chen et al., 2022). Tu-Fu-Ling could significantly downregulates the expression of renal inflammatory factors (Wang et al., 2019). *Mesona procumbens* Hemsl. could effectively relieve renal inflammation in STZ-induced diabetic rats (Jhang et al., 2016). *Chrysanthemum morifolium* Ramat. obviously attenuated renal inflammation (Peng et al., 2019).

### 6.2 Alleviation of renal oxidative stress

An *in vivo* study has illustrated that Tu-Fu-Ling could increase the activity of catalase and thus alleviate the oxidative stress in rat kidneys caused by hyperuricemia (Hong et al., 2014). In addition, Wang et al. (2019) proved that the flavonoid-rich fraction extracted from Tu-Fu-Ling could significantly attenuate damages in renal tubular epithelial cells and alleviate the renal oxidative stress. Meng et al. (2017) have found that baicalein extracted from Scutellariae radix could alleviate the tubulointerstitial damage and NADPH oxidase–dependent renal oxidative stress in HN mice.

### 6.3 Inhibition of renal fibrosis

Geniposide extracted from Fructus Gardenia and baicalein extracted from Scutellariae radix could significantly inhibit renal fibrosis (Meng et al., 2017; Chen et al., 2022). Moreover, the barks of *Liriodendron chinense* (Hemsl.) Sarg remarkedly inhibited renal fibrosis *via* JAK2/STAT3 signaling pathways (Pan et al., 2021).

## 7 Discussion

This review presented the results of the investigations on hypouricemic effects and renal-protective effects of traditional Chinese herbs conducted so far. Existing studies proved that the aforementioned 11 herbs could treat HN *via* lowering serum UA (by inhibiting liver XOR activity and regulating the expressions of renal urate transporters or both) and protecting renal function directly.

TCM always focuses on the clinical experiences of physicians. A large number of clinical studies have investigated the therapeutic effects of traditional Chinese herbs on hyperuricemia and hyperuricemia-induced CKD. A meta-analysis published in 2016 included 11 randomized controlled clinical trials with 838 patients and found the UA-lowering effects of traditional Chinese herbs were significantly superior to those of commercial drugs (RR: 1.11; 95% CI: 1.04–1.17; *p* = 0.0007) (Lin et al., 2016). Notably, the traditional Chinese herbs were better than commercial drugs in reducing adverse effects (RR: 0.30; 95% CI: 0.15–0.62; *p* = 0.001) (Lin et al., 2016). Although the efficacy of traditional Chinese herbs on hyperuricemic nephropathy is well-established, modern pharmacological studies were missing for a long period. With the development of TCM modernization, growing numbers of basic research studies explored the effects and underlying mechanisms of traditional Chinese herbs in animal models.

This review was conducted by searching major databases of published articles. After the systematic search of the literature, 11 traditional Chinese herbs were identified. All of them have both UA-lowering effects and renal-protective effects. Two herbs (*Smilax china* L. and Scutellariae radix) could inhibit liver XOR activity, three herbs (*Liriodendron chinense* (Hemsl.) Sarg, Poria cocos and Dendrobium officinalis six nostrum)could regulate expressions of renal urate transporters, five herbs could both inhibit liver XOR activity and regulate expressions of renal urate transporters, and one herb (*Rhododendron oldhamii* Maxim.) lowered serum UA with unclear mechanisms. When it comes to renal protection, six herbs could inhibit renal inflammation, three herbs could alleviate renal oxidative stress, and three herbs could inhibit renal fibrosis. Furthermore, the aforementioned 11 herbs have no apparent adverse reactions.

Notably, several limitations should be considered when applying traditional Chinese herbs in the treatment of HN. First, at present, the majority of basic research studies focused only on the evaluation of efficacy and the underlying mechanisms have not been thoroughly investigated. Second, it remains unclear whether the renal-protective effects of traditional Chinese herbs are UA-lowering effects dependent or not. Third, recently, integrated traditional Chinese and Western medicine therapy have been widely used in the treatment of multiple diseases. However, based on HN treatment, relevant high-quality studies are lacking. The combination of modern and traditional medicine could develop a new strategy to treat HN efficiently. Fourth, to some extent, rigorous large clinical trials are needed to confirm the efficiency and safety of traditional Chinese herbs or compounds.

In conclusion, traditional Chinese herbs have a good application prospect in the treatment of hyperuricemia-induced CKD. The detailed mechanism needs further investigation in the future.
